# Frontiers in Occupational Health and Safety Management

**DOI:** 10.3390/ijerph191710759

**Published:** 2022-08-29

**Authors:** Delfina Ramos, Teresa Cotrim, Pedro Arezes, João Baptista, Matilde Rodrigues, João Leitão

**Affiliations:** 1 School of Engineering, Polytechnic of Porto, ISEP, 4249-015 Porto, Portugal; 2 INEGI—Instituto de Ciência e Inovação em Engenharia Mecânica e Engenharia Industrial, 4200-465 Porto, Portugal; 3 Algoritmi Centre, School of Engineering, University of Minho, 4800-058 Guimarães, Portugal; 4 Ergonomics Laboratory, Faculdade de Motricidade Humana, Universidade de Lisboa, 1499-002 Cruz Quebrada, Portugal; 5 Research Centre for Architecture, Urbanism and Design (CIAUD), Faculdade de Arquitetura, Universidade de Lisboa, 1349-063 Lisboa, Portugal; 6 Associated Laboratory for Energy, Transports and Aeronautics, LAETA (PROA), Faculty of Engineering, University of Porto, 4200-465 Porto, Portugal; 7 Porto Biomechanics Laboratory, Faculty of Sport, University of Porto, 4200-450 Porto, Portugal; 8 Research Centre on Environment and Health, Department of Environmental Health, School of Health, Polytechnic Institute of Porto, 4200-072 Porto, Portugal; 9 NECE–Research Unit in Business Sciences, Faculty of Human and Social Sciences, University of Beira Interior (UBI), Estrada do Sineiro, 6200-001 Covilhã, Portugal; 10 Centre for Management Studies of Instituto Superior Técnico (CEG-IST), University of Lisbon, 1649-004 Lisboa, Portugal; 11 Instituto de Ciências Sociais (ICS), University of Lisbon, 1649-004 Lisboa, Portugal

## Overview

This Special Issue of the *International Journal of Environmental Research and Public Health* is devoted to the “Frontiers in Occupational Health and Safety Management”. This issue intends to contribute to the knowledge in the field regarding the new challenges for Occupational Safety and Health (OSH) management. This concern is stated in the EU Strategic Framework on Health and Safety at Work for 2021–2027, which sets out the key actions needed to improve the health and safety of workers in the years to come. This new strategy focuses on three cross-cutting objectives, namely, anticipating and managing change in the context of green, digital, and demographic transitions; improving the prevention of work-related accidents and diseases and striving towards a Vision Zero approach to work-related deaths; and increasing the preparedness to respond to current and future health crises [1]. This strategy is expected to have a significant impact on the management of OSH at all levels, including companies from all sectors. It is also a basis for increasing the awareness and funding support for the improvement of the health and safety of workers. The European Agency for Safety and Health at Work (EU-OSHA) has an important role in implementing this strategy at the European level, but also in coordination with the numerous National Agencies.

The COVID-19 pandemic has had a profound impact on nearly every aspect of the world of work. The frontiers of OSH were pushed when outbreaks of SARS-CoV-2 were observed in workplaces, exposing workers, their families, and communities to the risk of infection. In addition to the risk of infection, workers in all sectors face additional hazards that have emerged due to new work practices and procedures adopted to mitigate the spread of the virus. Teleworking, for example, has led to ergonomic and psychosocial risks with some 65% of surveyed companies reporting that workers’ morale has been difficult to sustain while working from home [2,3]. Additionally, self-reported symptoms of computer vision syndrome have increased in several settings [4]. The time spent by workers looking at the computer screen during the pandemic has increased due to the long working hours, thereby increasing eye and vision complaints. To face these problems, new guidance has emerged with respect to working safely during the COVID-19 pandemic, providing a set of guidelines to manage the risks that have been arising and to enable the effective and timely adaptation to changing situations (e.g., ISO/PAS 45005:2020 [5]).

The COVID-19 pandemic, by showing that organisations were not prepared to lead with this new risk, drew attention to emergency management, particularly for organizations in the field of public health. The International Labour Organization (ILO), on the World Day for Safety and Health at Work (28 April 2021), called on countries to implement resilient OSH systems for future health emergencies. This will require investments in OSH infrastructure and its integration into comprehensive national crisis emergency preparedness and response plans to protect the safety and health of workers and support business continuity. Thus, organizational resilience has been given a new boost [2].

Resilience can be defined as the process and outcome of successfully adapting to difficult or challenging life experiences, especially through mental, emotional, and behavioural flexibility and adjustment to external and internal demands. The concept of resilience has become very popular, especially in the 21st century. In the perspective of organisations, it becomes clear that they must be resilient to rapid technological, environmental, or other types of changes and install or remove controls quickly and efficiently. Therefore, dynamic decision making on risk controls will challenge the traditional strategies of OHS [6]. Risk management, also considered as uncertainty management, allows organisations to attempt to prepare for the unexpected by minimising risks and extra costs before they happen [7]. From the individual point of view, self-confidence is a good means to cope with the stresses of life and plays an important part in resilience. Becoming more confident in one’s personal abilities, including the ability to respond to and deal with a crisis, is a great way to build resilience for the future. This concept has become increasingly important with the COVID-19 crisis and has also a certain connection with OSH.

The industry revolution has completely changed the way work is executed. At the same time that it brought solutions for OSH, it also brought new risks that need to be recognized and minimized. The frontiers in OSH changed again with the concept of Industry 4.0, which is now evolving towards Industry 5.0. The human factor becomes the central axis for the formation of smart cyber-physical socio-technical systems that are integrated into workplaces’ physical and cultural host environments. New risks have emerged, leading to a potential transformation of OSH, and giving rise to a new model called OSH 5.0, in which innovation, digitalization, and cultural transformations constitute sources of value in work and in its development contexts, in parallel with the concept of Industry 5.0 [8]. Therefore, the frontiers of OSH are enlarged within this context.

There is a gradual transition to a circular economy, which is a key driver of the EU’s goal of achieving carbon neutrality by 2050 while creating sustainable growth and jobs. The circular economy has significant policy and regulatory implications that will affect future jobs. It will also have consequences for workers’ safety and health. There is, for instance, an impact on jobs in hazardous sectors related to maintenance and repair as well as disassembly and recycling, which can have a negative impact on working conditions [9]. The circular economy contributes to better and greater environmental sustainability and a better intervention at the social level [10]. The circular economy also leads to changes in organizational processes and/or redesigning tasks, which can have an impact on job content and satisfaction. The circular economy also influences the enlargement of the frontiers of OSH [9].

Technological advancement is often a double-edged sword in that it presents both risks and opportunities. The increasing use of artificial intelligence (AI) is continuously transforming jobs and work tasks. Although AI-based systems in the workplace offer many benefits, there is a growing debate on how they impact OSH. The automation (or semi-automation) of cognitive tasks in particular introduces concerns about workers’ psychosocial wellbeing that must be addressed by policymakers. The recent EU-OSHA report on ‘Artificial Intelligence and automation of cognitive tasks: Implications for occupational safety and health’ identifies a number of key risks that should be addressed by policymakers through analysing labour law and data protection regulation. The most obvious concern is the threat of job loss, followed by feelings of precarity at work and poor mental health. As intelligent programs more efficiently process forms, applications, claims, legal documents, etc., it will no longer be necessary for humans to complete these ‘mind-numbing’ and alienating tasks [11].

The impact of artificial intelligence on the workplace might create opportunities but also new challenges for OSH, its management, and its regulation. Artificial intelligence has also facilitated the emergence of new forms of monitoring and managing workers based on the collection of large amounts of real-time data. These novel forms may provide an opportunity to improve OSH surveillance, reduce exposure to various risk factors, and provide early warnings of stress, health problems, and fatigue. However, they might also give rise to legal, regulatory, and ethical questions, as well as concerns for OSH [12]. Again, Artificial Intelligence broadens the frontiers of OSH.

Big data refers to data sets that contain greater variety, arriving in increasing volumes and with greater velocity, as they are too large or complex to be dealt with by traditional data-processing application software. According to Wang and Wang [13], big data has an important influence on safety management in various fields where its applications are becoming more prevalent. The analysis results of big data have become an important reference influencing safety-related decision making.

The implementation of integrated management systems allows organizations to achieve efficient results in reducing risks and increasing productivity, providing a better understanding of how management systems influence the OSH risk management in organizations, particularly in SMEs. The success of the integration of risk management in OSH depends on both technical and human aspects [14].

In the last decade, there has been a rapid emergence of nanotechnology into several consumer products, which has led to concerns regarding the potential risks for human health following consumer exposure. There is also a concern in terms of occupational safety and health, related to the exposure of workers involved in the manufacturing, processing, and handling of consumer goods containing nanomaterials. Exposure to engineered nanomaterials has been associated with several health effects including pulmonary inflammation, genotoxicity, carcinogenicity, and circulatory effects. Textiles are one of the most heavily traded commodities in the world. The textile industry is already an important user of nanotechnologies and there are a significant number of “nanotextiles” in the market, including many consumer skin-contact goods, which have been introduced by the incorporation of nanoparticles [15]. The risk for the workers and for the consumers is linked to the characteristic properties of nanomaterials that make them different from their macroscale counterparts and are determined by the physicochemical properties of the nanomaterial, the interactions with the materials, and the potential exposure levels. The growing concern about the possible negative effects of nanomaterials on humans and on the environment can lead to restrictions for consumer products incorporating nanoparticles. There are many studies about the penetration of nanoparticles into the skin, related, for instance, to sunscreens and cosmetics, which are often based on nanomaterials. In fact, only the smaller nanoparticles seem to be able to penetrate the undamaged skin, although if the skin is injured, larger nanoparticles can penetrate [15]. Nanotechnology is no doubt a concern and broadens the frontiers of OSH.

Industrial intelligence is a global trend. In developed countries, industrial intelligence has been developing under relatively mature economic and technology conditions, known as a tech-led pattern. In other words, industrial intelligence occurs naturally under the mature conditions of economic and technological conditions. In addition, one of the most critical conditions for industrial intelligence involves the skills of workers. Recent research argues that industrial intelligence creates better technology that reduces occupational injuries [16].

Recent research argues that robots could replace workers in dangerous work environments, for instance, in chemical and mining industries, with a strong impact on the reduction of occupational injuries [16,17]. However, according to Yang et al. [18], robot applications do not have a persistent impact on occupational injuries and can even increase the rate of occupational injuries in developing countries.

Concerning the more familiar occupational injuries, as outlined by Leitão et al. [19], there is still room for addressing the traditional motivators and hygiene factors associated with the promotion of the quality of working life (QWL), such as having an appropriate salary, having a safe work environment, and benefiting from occupational healthcare; thus, increased attention should be devoted to burnout factors, the so-called de-motivators, as a moderator of the relationship between QWL and the contribution to productivity at the organisational level. In this scope, the intelligent and learning algorithms and technologies could play a preventative role.

Implementing Industry 4.0 and interconnected robotization in industrial enterprises promotes occupational changes. It is essential to develop cooperation and collaboration between a robot and a human in a common robotized workplace so that robotization is safe and effective. A robotic device that works in collaboration with a human operator is called a cobot. Workplace robotization is particularly suitable for work environments that involve hazardous chemical substances that are carcinogenic and toxic to humans. Robotization also helps to improve workplace ergonomics and to avoid, for humans, very laborious and often repetitive work [17]. The automation of tasks with robots can remove workers from hazardous situations, and cobots can facilitate access to work for aging workers or those with disabilities [12]. The use of robots also presents a broadening of the frontiers of OSH.
